# Kinesin-1 autoinhibition facilitates the initiation of dynein cargo transport

**DOI:** 10.1083/jcb.202205136

**Published:** 2022-12-16

**Authors:** Rongde Qiu, Jun Zhang, Xin Xiang

**Affiliations:** 1 Department of Biochemistry and Molecular Biology, The Uniformed Services University of the Health Sciences, F. Edward Hébert School of Medicine, Bethesda, MA, USA

## Abstract

The functional significance of Kinesin-1 autoinhibition has been unclear. Kinesin-1 transports multiple cargoes including cytoplasmic dynein to microtubule plus ends. From a genetic screen for *Aspergillus* mutants defective in dynein-mediated early endosome transport, we identified a kinesin-1 mutation *kinA*^K895^* at the C-terminal IAK motif involved in autoinhibition. The *kinA*^∆IAK^ and *kinA*^K895E^ mutants exhibited a similar defect in dynein-mediated early endosome transport, verifying the importance of kinesin-1 autoinhibition in dynein-mediated transport. Kinesin-1 autoinhibition is not critical for dynein accumulation at microtubule plus ends or for the secretory vesicle cargoes of kinesin-1 to reach the hyphal tip. However, it facilitates dynein to initiate early endosome transport. This is unrelated to a direct competition between dynein and kinesin-1 on early endosomes because kinesin-3 rather than kinesin-1 drives the plus-end-directed early endosome movement. This effect of kinesin-1 autoinhibition on dynein-mediated early endosome transport is related to cargo adapter-mediated dynein activation but at a step beyond the switching of dynein from its autoinhibited conformation.

## Teaser

A screen for mutants defective in dynein-mediated early endosome transport identified a kinesin-1 mutation that disrupts kinesin-1 autoinhibition. Kinesin-1 autoinhibition is not needed for driving dynein accumulation at microtubule plus ends, but it affects the initiation of dynein-mediated cargo transport.

## Introduction

Kinesin-1s are plus-end-directed microtubule motors that transport various cargoes, and kinesin-1 defects cause many human diseases (Boecker et al., 2021; Brenner et al., 2018; Gennerich and Vale, 2009; Hannaford et al., 2022; Hirokawa and Tanaka, 2015; Kelliher et al., 2018; Keren-Kaplan and Bonifacino, 2021; Kruppa and Buss, 2021; Nicolas et al., 2018; Roney et al., 2021). The activity of kinesin-1 is regulated by multiple factors, including its light chains, cargo adapter proteins, an opposite motor that binds to the same cargo, phosphorylation, and microtubule-binding proteins (Ally et al., 2009; Balabanian et al., 2022; Blasius et al., 2007; Byrd et al., 2001; Chen and Sheng, 2013; Fenton et al., 2021; Ferro et al., 2022; Fu and Holzbaur, 2013; Gindhart et al., 1998; Guardia et al., 2021; Keren-Kaplan and Bonifacino, 2021; Monroy et al., 2020; Sakamoto et al., 2005; Twelvetrees et al., 2019; Verhey and Hammond, 2009; Williams et al., 2014; Xu et al., 2012; Zhao et al., 2021). An important aspect of kinesin-1 regulation is its autoinhibition that involves an interaction between the C-terminal tails and motor domains (Cai et al., 2007; Chiba et al., 2022; Coy et al., 1999; Dietrich et al., 2008; Friedman and Vale, 1999; Kaan et al., 2011; Seiler et al., 2000; Stock et al., 1999; Weijman et al., 2022; Wong et al., 2009). Recently, a KIF5A (kinesin-1) mutation causing the neurodegenerative disease Amyotrophic Lateral Sclerosis (ALS) has been linked to a loss of autoinhibition (Baron et al., 2022; Nakano et al., 2022; Pant et al., 2022). However, the importance of kinesin-1 autoinhibition in normal cells needs to be dissected.

Cytoplasmic dynein is the minus-end-directed microtubule motor transporting many cargoes (Reck-Peterson et al., 2018), and defects in dynein and/or its regulator dynactin are implicated in ALS or ALS-like motor neuron degeneration (Chevalier-Larsen and Holzbaur, 2006; Gershoni-Emek et al., 2016; Hafezparast et al., 2003; Ikenaka et al., 2013; Kieran et al., 2005; Lai et al., 2007; LaMonte et al., 2002; Maimon et al., 2021; Mentis et al., 2022; Münch et al., 2004; Stavoe and Holzbaur, 2019; Ström et al., 2008; Yu et al., 2018). Some vesicular cargoes bind dynein and kinesin-1 through the same cargo adapter (Celestino et al., 2022; Cox and Spradling, 2006; Fenton et al., 2021; Fu and Holzbaur, 2014; Canty et al., 2021 *Preprint*), and in this case, loss of kinesin-1 autoinhibition may overcome dynein to change cargo distribution (Baron et al., 2022; Kelliher et al., 2018). In many cell types, dynein itself is a cargo of kinesin-1 (Arimoto et al., 2011; Brendza et al., 2002; Duncan and Warrior, 2002; Egan et al., 2012; Hirokawa et al., 1990; Januschke et al., 2002; Lenz et al., 2006; Twelvetrees et al., 2016; Yamada et al., 2010; Zhang et al., 2003). In mammalian neurons, kinesin-1 interacts with dynein directly through kinesin light chains and moves dynein towards microtubule plus ends (Ligon et al., 2004; Twelvetrees et al., 2016). In filamentous fungi, kinesin-1s are important (although not essential) for the microtubule plus-end accumulation of dynein and dynactin (Egan et al., 2012; Lenz et al., 2006; Peñalva et al., 2017; Yao et al., 2012; Zhang et al., 2003; Zhang et al., 2010). Fungal kinesin-1s do not have associated light chains (Seiler et al., 2000; Steinberg and Schliwa, 1995), but dynactin is important for the dynein-kinesin-1 interaction (Qiu et al., 2018). The kinesin-1-mediated dynein accumulation at the plus ends is important for the retrograde transport of dynein cargoes such as early endosomes (Abenza et al., 2009; Lenz et al., 2006; Zekert and Fischer, 2009). Plus-end-directed transport of early endosomes does not use kinesin-1 but uses kinesin-3 (Lenz et al., 2006; Wedlich-Söldner et al., 2002; Zekert and Fischer, 2009). Dynein transports early endosomes away from the plus ends with the help of dynactin and the FTS-Hook-FHIP complex (Bielska et al., 2014; Yao et al., 2014; Zhang et al., 2011b; Zhang et al., 2014). The mechanism of dynein-mediated early endosome transport is largely conserved and has been dissected in detail using mammalian proteins (Christensen et al., 2021; Guo et al., 2016; Lau et al., 2021; Olenick et al., 2019; Schroeder and Vale, 2016; Urnavicius et al., 2018; Yeh et al., 2012). However, factors regulating this process require further studies. Here, we present our work identifying kinesin-1 autoinhibition as a new factor important for the initiation of dynein-mediated early endosome transport.

## Results and discussion

### Identifying the *kinA*^K895^* mutation affecting dynein-mediated early endosome transport

**Figure 1.**
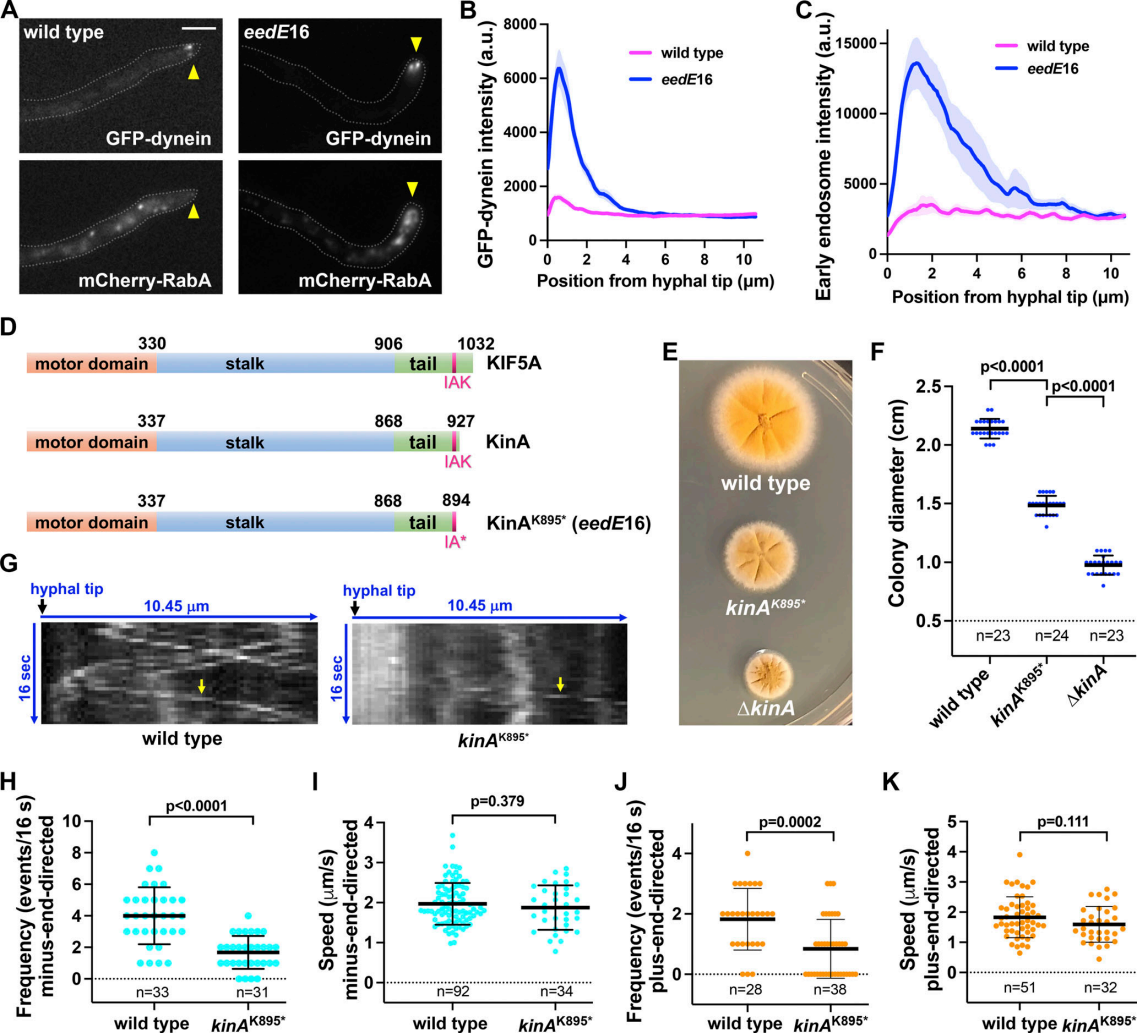
**Phenotype of the *eedE*16 (*kinA***^K895^*) mutant and position of the *kinA*^K895^* mutation in KinA kinesin-1. (A) Microscopic images showing the distributions of GFP-dynein and mCherry-RabA-labeled early endosomes (mCherry-RabA) in wild type and the mutant. Bar, 5 μm. Although bi-directional movements of mCherry-RabA-labeled early endosomes are not completely abolished, most of the *eedE*16 hyphal tips (∼80%) show an obvious accumulation of mCherry-RabA signals (*n* = 130). Hyphal tip is indicated by a yellow arrowhead. **(B)** Line scans of GFP-dynein fluorescence intensity in wild type and the mutant. XY graphs with mean (solid lines) and SEM (shading) were generated by Prism 9. GFP-dynein intensity near the hyphal tip (between 0 and 2.145 μm from hyphal tip) was significantly higher in the mutant than in wild type (P < 0.0001, two-way ANOVA with Bonferroni’s multiple comparisons test, *n* = 30 hyphae for wild type, *n* = 30 hyphae for the mutant). **(C)** Line scans of mCherry-RabA (early endosomes) fluorescence intensity in wild type and the mutant. XY graphs with mean (solid lines) and SEM (shading) were generated by Prism 9. The intensity of mCherry-RabA near the hyphal tip (between 0.585 and 2.730 μm from hyphal tip) was significantly higher in the mutant than in wild type (P < 0.0001, two-way ANOVA with Bonferroni’s multiple comparisons test, *n* = 30 hyphae for wild type, *n* = 30 hyphae for the mutant). **(D)** Domain structures of kinesin-1 proteins in human (KIF5A) and *A. nidulans* (KinA). Positions of the IAK motif involved in kinesin-1 autoinhibition and the *eedE*16 mutation *kinA*^K895*^ within the IAK motif are indicated. **(E)** Colony phenotypes of a wild-type strain, the *kinA*^K895^* mutant and the ∆*kinA* mutant. **(F)** A quantitative analysis of colony diameter of the wild-type strain (*n* = 23), the *kinA*^K895^* mutant (*n* = 24) and the ∆*kinA* mutant (*n* = 23; ordinary one-way ANOVA with Tukey’s multiple comparisons test). Scatter plots with mean and SD values were generated by Prism 9. **(G)** Kymographs showing mCherry-RabA signals (diagonal lines indicating movements of mCherry-RabA-marked early endosomes). Yellow arrows indicate dynein-mediated (or minus-end-directed) movements away from the hyphal tip. **(H)** A quantitative analysis on the frequency of minus-end-directed early endosome transport in wild type (*n* = 33 hyphal tips) and the *kinA*^K895^* mutant (*n* = 31 hyphal tips; unpaired *t* test, two-tailed, Prism 9). Scatter plots with mean and SD values were generated by Prism 9. **(I)** A quantitative analysis on the speed of minus-end-directed transport in wild type (*n* = 92 movements) and the *kinA*^K895^* mutant (*n* = 34 movements; unpaired *t* test, two-tailed, Prism 9). **(J)** A quantitative analysis on the frequency of plus-end-directed early endosome transport in wild type (*n* = 28 hyphal tips) and the *kinA*^K895^* mutant (*n* = 38 hyphal tips; unpaired *t* test, two-tailed, Prism 9). Scatter plots with mean and SD values were generated by Prism 9. **(K)** A quantitative analysis on the speed of plus-end-directed transport in wild type (*n* = 51 movements) and the *kinA*^K895^* mutant (*n* = 32 movements; unpaired *t* test, two-tailed, Prism 9).

In *Aspergillus nidulans*, microtubule plus ends face the hyphal tip, and minus ends are at either the spindle-pole body or septum (Efimov et al., 2006; Egan et al., 2012; Han et al., 2001; Oakley et al., 1990; Xiong and Oakley, 2009; Zeng et al., 2014; Zhang et al., 2017b). The plus-end accumulation of dynein is represented by the comet-like structures of GFP-dynein near the hyphal tip (Han et al., 2001; Xiang et al., 2000). A defect in dynein-mediated transport causes an abnormal hyphal-tip accumulation of early endosomes and their hitchhiking cargoes (Abenza et al., 2009; Lenz et al., 2006; Salogiannis and Reck-Peterson, 2017; Zekert and Fischer, 2009). Genetic screens for early-endosome distribution (*eed*) mutants allowed the identification of novel dynein regulators (Xiang and Qiu, 2020). Here, we performed a UV mutagenesis using a strain containing mCherry-RabA-marked early endosomes and GFP-labeled dynein heavy chain (Pinar and Peñalva, 2021; Zhuang et al., 2007). In the *eedE*16 mutant isolated after mutagenesis, we observed plus-end GFP-dynein comets at the hyphal tip (Fig. 1 A). Diffuse signals near the comets possibly contributed to an increase in the hyphal-tip GFP-dynein signal intensity (Fig. 1, A and B). Early endosomes (mCherry-RabA) abnormally accumulated near the hyphal tip (Fig. 1, A and C).

**Figure S1.**
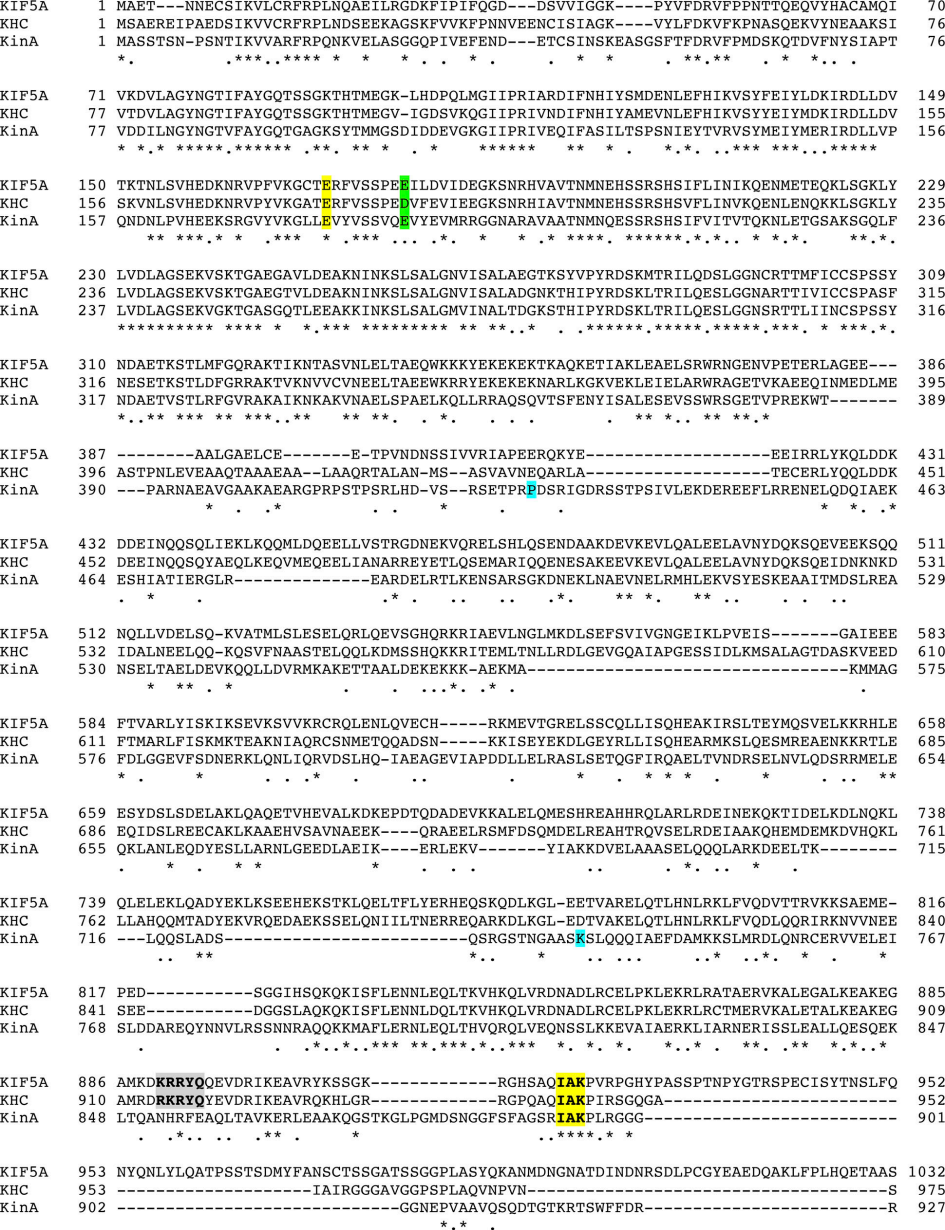
**A sequence alignment of kinesin-1s including KIF5A, kinesin-1 heavy chain in Drosophila (KHC) and kinesin-1 in *A. nidulans* (KinA).** The alignment was done using MacVector T-Coffee multiple sequence alignment (Pairwise Mode: Myers Miller). Residues that are identical (*), strongly similar (:) or weakly similar (.) are indicated. The IAK motif is highlighted in yellow and the C-terminal microtubule-binding domain is shaded with gray. E178 (KinA) is highlighted in yellow, E186 in green, P426 in blue, and K735 in blue.

The *eedE16* mutation was identified through whole-genome sequencing, and it is in *kinA*, which encodes the only kinesin-1 in *A. nidulans* (Requena et al., 2001). The mutation substituted an A with T that changed the codon for lysine (K; AAG) to a stop codon (TAG) at residue 895 of KinA, a 927-aa protein. This results in the deletion of 33 amino acids at the C-terminus of KinA. The C-termini of *Drosophila* and human Kinesin-1s contain two important regions: the IAK motif involved in autoinhibition (Kaan et al., 2011), and the RKRYQ region responsible for ATP-independent microtubule binding (Lu et al., 2016; Winding et al., 2016). The IAK motif but not the RKRYQ region is conserved in KinA (Fig. S1). K895 corresponds to K within the IAK motif (Fig. 1 D and Fig. S1). Since this motif is key to autoinhibition while additional C-terminal residues may further stabilize the autoinhibited structure (Hackney and Stock, 2000; Kaan et al., 2011), the *kinA*^K895^* mutation may eliminate kinesin-1 autoinhibition.

The phenotype of the *kinA*^K895^* mutant differs from that of the ∆*kinA* mutant (Requena et al., 2001). Loss of *kinA* causes dynein to localize along microtubules and decreases its plus-end accumulation near hyphal tip (Egan et al., 2012; Peñalva et al., 2017; Zhang et al., 2010), but *kinA*^K895^* causes a stronger hyphal-tip dynein accumulation (Fig. 1, A and B). The *kinA*^K895^* mutant colony is smaller than a wild-type colony but significantly bigger than the ∆*kinA* colony (Fig. 1, E and F). Thus, the normal kinesin-1 function is partially retained in the *kinA*^K895^* mutant.

**Figure S2.**
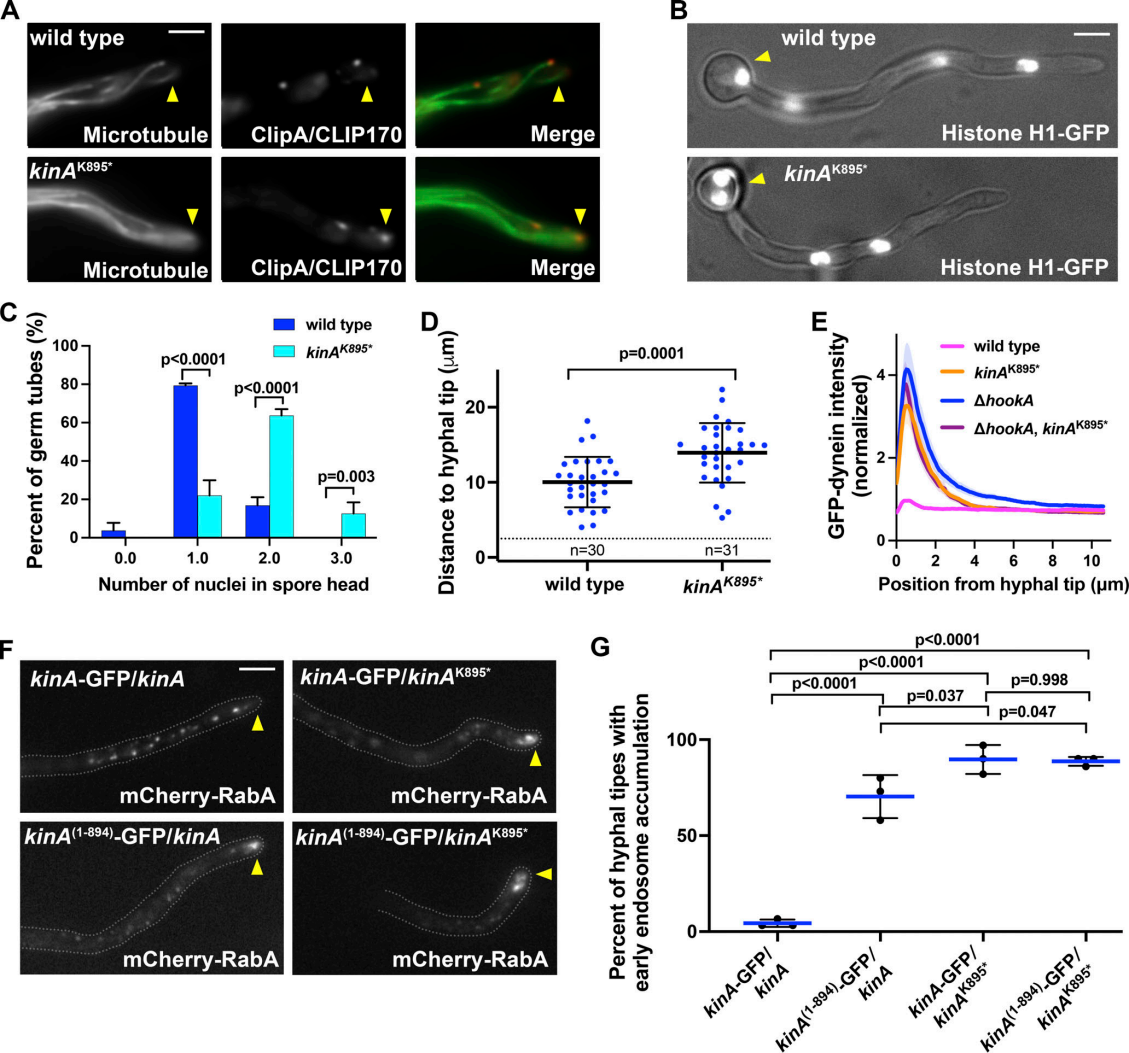
**Phenotypes of the *kinA***^K895^* **mutant and various diploids. (A)** Images of GFP-TubA (Microtubule; Han et al., 2001) and mCherry-ClipA (ClipA/CLIP170; Zeng et al., 2014) in wild type and the *kinA*^K895^* mutant, showing normal microtubule polarity in the *kinA*^K895^* mutant. Hyphal tip is indicated by a yellow arrowhead. Bar, 5 μm. Note that 21 randomly chosen wild-type hyphae all show normal microtubule polarity, and 93.3% of 45 randomly chosen *kinA*^K895^* mutant hyphae show normal microtubule polarity indicated by microtubule plus ends pointing towards the hyphal tip. In the small number of hyphal tips where we observed microtubule plus ends pointing away from the hyphal tip, the microtubule polarity might still be correct but these microtubules appeared to curl around the hyphal tip, possibly due to an increase in microtubule stability. **(B)** Images of germ tubes showing nuclei labeled with histone H1-GFP (Xiong and Oakley 2009) in wild type and the *kinA*^K895^* mutant. The spore head is indicated by a yellow arrowhead. Bar, 5 μm. **(C)** A quantitative analysis on the percent of germ tubes containing different numbers of nuclei in the spore head. Column bar graphs with mean and SD values were generated from three experiments (for each experiment, at least 30 hyphae from each strain were counted, and the total n number for wild type is 164, and that for the mutant is 195). Significantly more wild-type germ tubes contain one nucleus in the spore head, while significantly more *kinA*^K895^* germ tubes contain two nuclei in the spore head (P < 0.0001 in both cases, two-way ANOVA with Bonferroni’s multiple comparisons test). **(D)** A quantitative analysis on the distance from the hyphal tip to its most proximal nucleus (*n* = 30 for wild type and *n* = 31 for the *kinA*^K895^* mutant, unpaired *t* test, two-tailed, Prism 9). **(E)** Line scans of GFP-dynein fluorescence intensity in wild type, the *kinA*^K895^* single mutant, the ∆*hookA* single mutant, and the *kinA*^K895^*, ∆*hookA* double mutant. XY graphs with mean (solid lines) and SEM (shading) were generated by Prism 9. All values are relative to the peak mean value for wild type, which is set as 1. GFP-dynein intensity near the hyphal tip (between 0.130 and 1.625 μm from hyphal tip) was significantly higher in the *kinA*^K895^* single mutant, the ∆*hookA* single mutant or the *kinA*^K895^*, ∆*hookA* double mutant than in wild type (P < 0.0001, two-way ANOVA with Tukey’s multiple comparisons test, *n* = 40 hyphae for all strains). **(F)** Microscopic images showing the distributions of mCherry-RabA-labeled early endosomes in four different diploids. Hyphal tip is indicated by a yellow arrowhead. Bar, 5 μm. **(G)** A quantitative analysis on the percentage of hyphal tips with the abnormal accumulation of early endosomes. Three experiments were performed, and in each experiment, 30 or more hyphal tips were examined for each strain. Scatter plots with mean and SD values were generated by Prism 9 (the *P* values were generated from ordinary one-way ANOVA, unpaired).

Since microtubule polarity looked normal in the *kinA*^K895^* mutant (Fig. S2 A; and Videos 1 and 2), the hyphal-tip accumulation of early endosomes indicates a dynein defect. Our quantitation showed a significant decrease in the frequency but not the speed of dynein-mediated transport in the mutant (Fig. 1, G–I). The frequency but not the speed of plus-end-directed transport is also lowered (Fig. 1, J and K), although the movement is driven by kinesin-3 rather than kinesin-1 (Lenz et al., 2006; Wedlich-Söldner et al., 2002; Zekert and Fischer, 2009). This has been similarly observed in other dynein-pathway mutants (Egan et al., 2012; Zhang et al., 2011b, 2018), which is likely caused by kinesin-3 having a lower chance to meet the cargoes abnormally blocked at the hyphal tip. The *kinA*^K895^* mutation also causes a mild but noticeable nuclear-distribution defect (Fig. S2, B, C, and D), consistent with an effect on the function of dynein in nuclear movement (Xiang, 2018).

**Video 1.** **Microtubules (labeled by GFP-TubA) in a wild-type hypha.** Hyphal tip is indicated by a yellow arrow. An inverted fluorescence microscope (Nikon Ti2-E) was used for capturing images. 60 frames were taken with a 0.04-s exposure time and a 1-s interval between frames. Binning is 1 × 1.

**Video 2.** **Microtubules (labeled by GFP-TubA) in a *kinA***^K895^* mutant hypha. Hyphal tip is indicated by a yellow arrow. An inverted fluorescence microscope (Nikon Ti2-E) was used for capturing images. 60 frames were taken with a 0.04-s exposure time and a 1-s interval between frames. Binning is 1 × 1.

### The effect of the *kinA*^K895^* mutation is largely due to a loss of IAK-mediated autoinhibition

**Figure 2.**
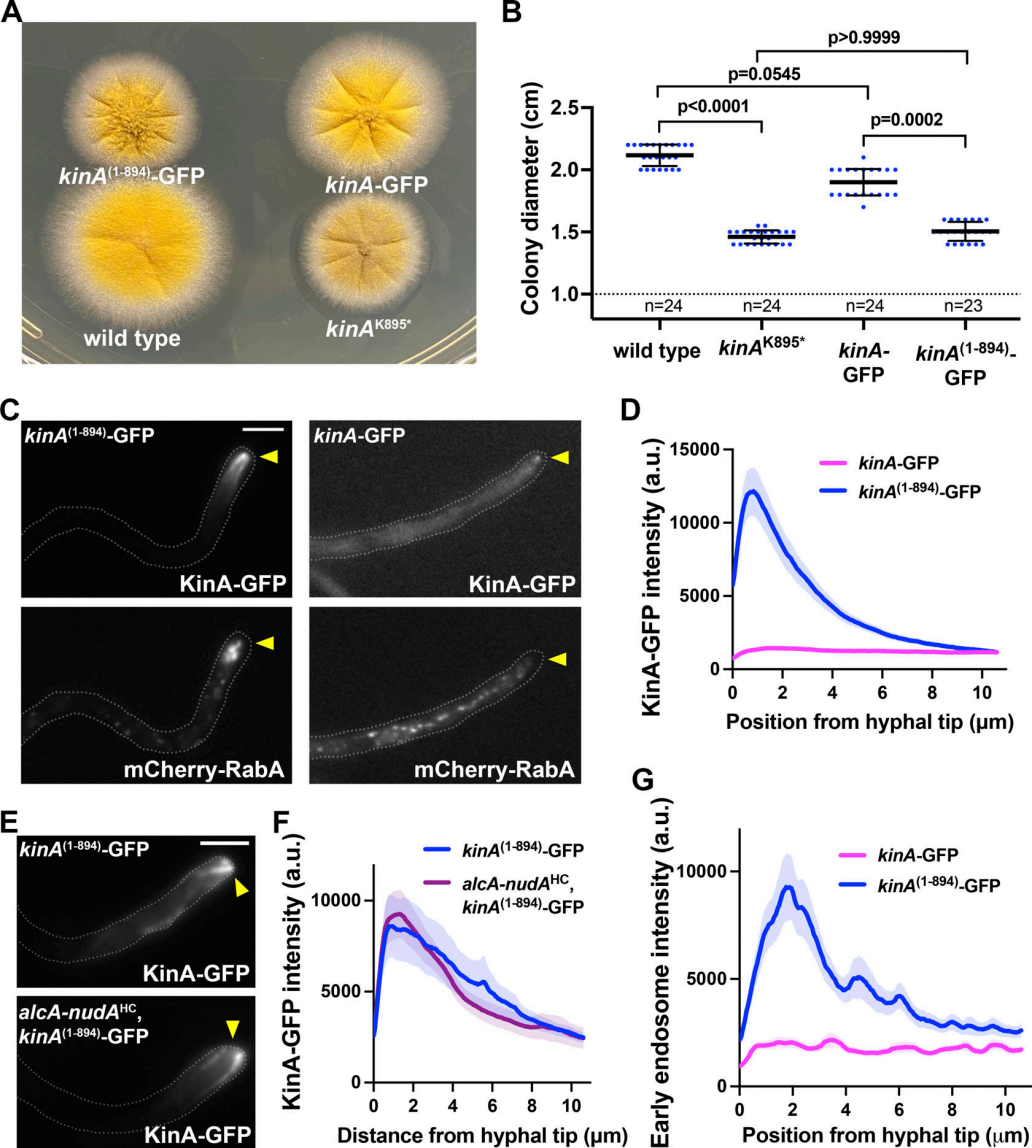
**KinA**^**(1–894)**^**-GFP fusion proteins form a prominent accumulation near the microtubule plus ends. (A)** Colony phenotypes of the strains containing *kinA*-GFP and *kinA*^(1–894)^-GFP in comparison to wild type and the *kinA*^K895^* mutant. **(B)** A quantitative analysis of colony diameter of the wild type (*n* = 24), *kinA*^K895^* (*n* = 24), *kinA*-GFP (*n* = 24) and *kinA*^(1–894)^-GFP (*n* = 23) strains (Kruskal–Wallis ANOVA test (unpaired) with Dunn’s multiple comparisons test). Scatter plots with mean and SD values were generated by Prism 9. **(C)** Localization of KinA-GFP or KinA^(1–894)^-GFP and mCherry-RabA-labeled early endosomes in strains containing one of the GFP fusions. Hyphal tip is indicated by a yellow arrowhead. Bar, 5 μm. **(D)** Line scans of KinA-GFP fluorescence intensity in the *kinA*-GFP (*n* = 33 hyphae) and *kinA*^(1–894)^-GFP (*n* = 32 hyphae) strains. XY graphs with mean (solid lines) and SEM (shading) were generated by Prism 9. The GFP intensity was significantly higher in the *kinA*^(1–894)^-GFP strain than in the *kinA*-GFP strain near the hyphal tip (between 0 and 3.51 μm from hyphal tip; P < 0.0001, two-way ANOVA with Bonferroni’s multiple comparisons test). **(E)** The hyphal-tip localization of KinA^(1–894)^-GFP in the *alcA-nudA*^HC^ conditional-null mutant. Images were taken from cells grown on glucose, a repressive medium for the regulatable *alcA* promoter, which allows the expression of the *nudA* gene (encoding dynein heavy chain) to be turned off. Hyphal tip is indicated by a yellow arrowhead. Bar, 5 μm. **(F)** Line scans of KinA-GFP fluorescence intensity in the *kinA*^(1–894)^-GFP strain (*n* = 20 hyphae) and the *alcA-nudA*^HC^, *kinA*^(1–894)^-GFP strain (*n* = 20). XY graphs with mean (solid lines) and SEM (shading) were generated by Prism 9. The intensity of KinA-GFP between 0 and 11 μm from hyphal tip was not significantly different in these two strains (P > 0.9999, two-way ANOVA with Bonferroni’s multiple comparisons test). **(G)** Line scans of mCherry-RabA (early endosomes) fluorescence intensity in the *kinA*-GFP (*n* = 33 hyphae) and *kinA*^(1–894)^-GFP (*n* = 32 hyphae) strains. XY graphs with mean (solid lines) and SEM (shading) were generated by Prism 9. The intensity of mCherry-RabA near the hyphal tip (between 0.715 and 3.12 μm from hyphal tip) was significantly higher in the *kinA*^(1–894)^-GFP strain than in the *kinA*-GFP strain (P < 0.0001, two-way ANOVA with Bonferroni’s multiple comparisons test).

To compare the wild-type and *kinA*^K895^* kinesin-1s, we replaced the wild-type *kinA* gene with either the *kinA*-GFP or *kinA*^(1–894)^-GFP fusion gene. The *kinA*^(1–894)^-GFP mutant formed a colony similar to that of the *kinA*^K895^* mutant (Fig. 2, A and B). KinA-GFP formed a diffuse background in the cytoplasm with occasional faint hyphal-tip signals, consistent with its autoinhibition, but KinA^(1–894)^-GFP formed a strong hyphal-tip accumulation at and/or near microtubule plus ends (Fig. 2, C and D), consistent with the localization of uninhibited kinesin-1s in other cell types (Baron et al., 2022; Seiler et al., 2000; Twelvetrees et al., 2016; Weijman et al., 2022). This accumulation was not affected by the lack of dynein heavy chain (*alcA-nudA*^HC^; Fig. 2, E and F). In the *kinA*^(1–894)^-GFP mutant, early endosomes (mCherry-RabA) accumulated abnormally near the hyphal tip (Fig. 2, C and G), just like in the *kinA*^K895^* mutant (Fig. 1, A and C).

**Figure 3.**
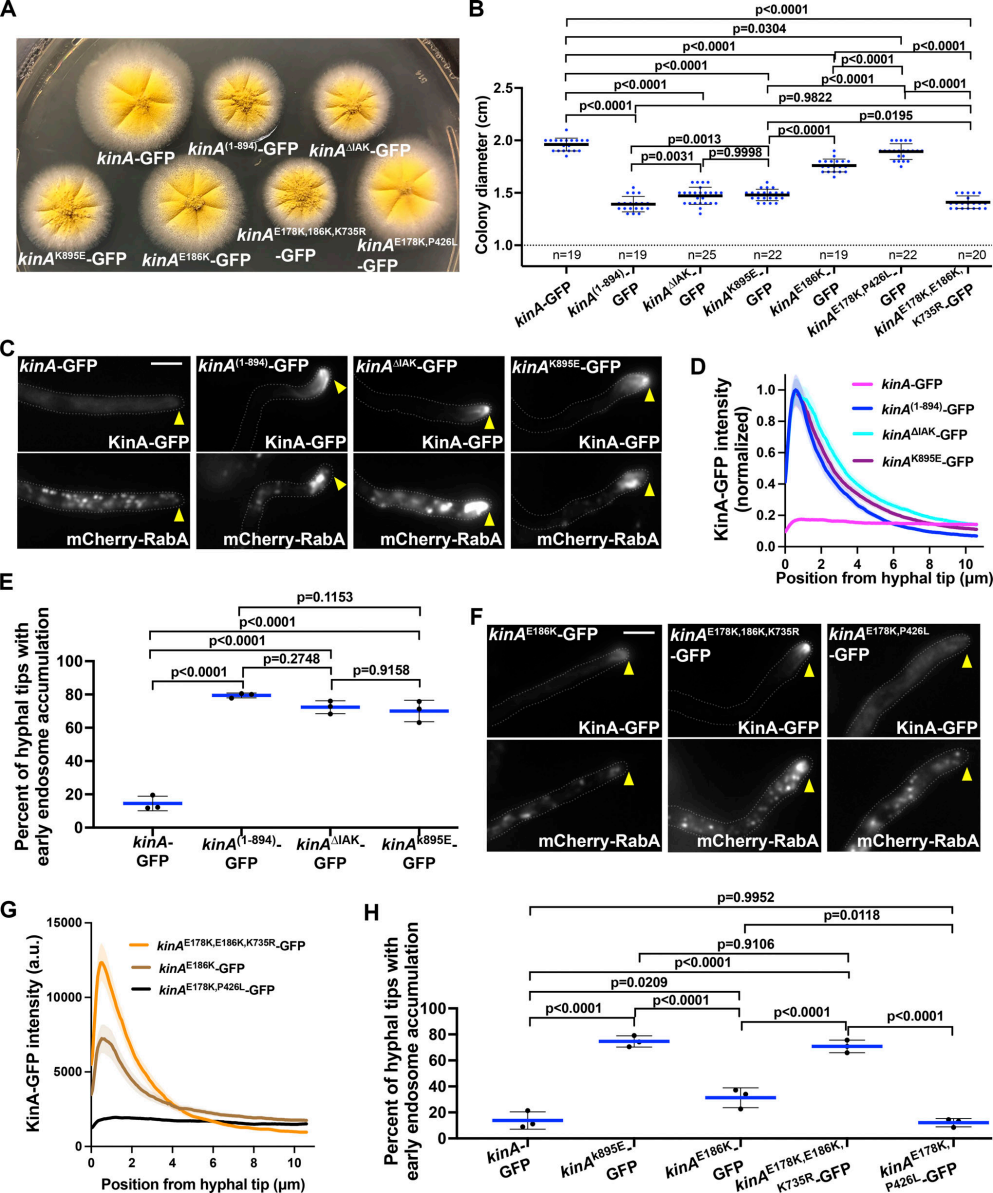
**Effects of kinesin-1 mutations implicated in autoinhibition on dynein-mediated early endosome transport. (A)** Colony phenotypes of the *kinA*-GFP, *kinA*^(1–894)^-GFP, *kinA*^∆IAK^-GFP, *kinA*^K895E^-GFP, *kinA*^E186K^-GFP, *kinA*^E178K,186K,K735R^-GFP, and *kinA*^E178K,P426L^-GFP strains. **(B)** A quantitative analysis of colony diameter of the *kinA*-GFP (*n* = 19), *kinA*^(1–894)^-GFP (*n* = 19), *kinA*^∆IAK^-GFP (*n* = 25), *kinA*^K895E^-GFP (*n* = 22), *kinA*^E186K^-GFP (*n* = 19), *kinA*^E178K,P426L^-GFP (*n* = 22), and *kinA*^E178K,186K,K735R^-GFP (*n* = 20) strains. Ordinary one-way ANOVA (unpaired) with Tukey’s multiple comparisons test was used to analyze these data sets. Scatter plots with mean and SD values were generated by Prism 9. **(C)** Microscopic images showing the distributions of KinA-GFP and mCherry-RabA-labeled early endosomes (mCherry-RabA) in the *kinA*-GFP, *kinA*^(1–894)^-GFP, *kinA*^∆IAK^-GFP, and *kinA*^K895E^-GFP strains. Bar, 5 μm. **(D)** Line scans of KinA-GFP fluorescence intensity in the *kinA*-GFP (*n* = 31), *kinA*^(1–894)^-GFP (*n* = 61), *kinA*^∆IAK^-GFP (*n* = 61) and *kinA*^K895E^-GFP (*n* = 60) strains. XY graphs with mean (solid lines) and SEM (shading) were generated by Prism 9. All values are relative to the peak mean value for *kinA*^(1–894)^-GFP, which is set as 1. The intensity of GFP near the hyphal tip (between 0 and 2.6 μm from hyphal tip) was significantly higher in the *kinA*^(1–894)^-GFP, *kinA*^∆IAK^-GFP or *kinA*^K895E^-GFP strain than in the *kinA*-GFP strain (P < 0.0001, two-way ANOVA with Tukey’s multiple comparisons test). **(E)** A quantitative analysis on the percentage of hyphal tips with the abnormal accumulation of early endosomes (Ordinary one-way ANOVA, unpaired). Three experiments were performed, and in each experiment, >80 hyphal tips were examined for each strain. Scatter plots with mean and SD values were generated by Prism 9. **(F)** Microscopic images showing the distributions of KinA-GFP and mCherry-RabA-labeled early endosomes (mCherry-RabA) in the *kinA*^E186K^-GFP, *kinA*^E178K,186K,K735R^-GFP, and *kinA*^E178K,P426L^-GFP strains. Bar, 5 μm. **(G)** Line scans of KinA-GFP fluorescence intensity in the *kinA*^E186K^-GFP (*n* = 30), *kinA*^E178K,186K,K735R^-GFP (*n* = 31), and *kinA*^E178K,P426L^-GFP (*n* = 30) strains. XY graphs with mean (solid lines) and SEM (shading) were generated by Prism 9. The intensity of GFP near the hyphal tip (between 0.065 and 1.885 μm from hyphal tip) was significantly different from each other among the three strains (P < 0.0001, two-way ANOVA with Tukey’s multiple comparisons test). **(H)** A quantitative analysis on the percentage of hyphal tips with the abnormal accumulation of early endosomes (Ordinary one-way ANOVA (unpaired) with Tukey’s multiple comparisons test). Three experiments were performed, and in each experiment, >40 hyphal tips were examined for each strain. Scatter plots with mean and SD values were generated by Prism 9.

To determine whether the defect in dynein-mediated early endosome transport is caused by a loss of IAK-mediated autoinhibition or by the loss of other deleted C-terminal amino acids that may regulate dynein, we made the *kinA*^∆IAK^-GFP and the *kinA*^K895E^-GFP mutants. These mutants looked similar to the *kinA*^(1–894)^-GFP mutant on plates (although their colonies are slightly bigger; Fig. 3, A and B), and they also exhibited a strong hyphal-tip accumulation of KinA-GFP (Fig. 3, C and D). Importantly, early endosomes accumulated abnormally near the hyphal tip in these mutants (Fig. 3, C and E), indicating that the IAK motif plays a key role in dynein-mediated early endosome transport.

Several amino acids in the motor domain of Drosophila KHC have been implicated in autoinhibition (Kaan et al., 2011; Kelliher et al., 2018). For example, D185 may interact with the IAK motif (Kaan et al., 2011), and E177K relieves autoinhibition in neurons (Kelliher et al., 2018). Since E186 of KinA corresponds to D185 of Drosophila KHC (Fig. S1), we constructed the *kinA*^E186K^-GFP strain. We also tried to construct a *kinA*^E178K^-GFP mutant (corresponding to E177K in Drosophila KHC; Fig. S1) as well as a *kinA*^E178K,E186K^-GFP mutant. However, the *kinA*^E178K^-GFP mutant we intended to make contained an extra mutation (P426L), and the *kinA*^E178K,E186K^-GFP mutant also contained an extra mutation (K735R). As the extra mutation in either case is in a non-conserved amino acid (Fig. S1), and K735R does not change the charge, we studied all these mutants further. Interestingly, E178K had almost no effect (Fig. 3, A, B, and F–H), possibly due to KinA being subtly different from Drosophila KHC in structure. While E186K only had a moderate effect (Fig. 3, A, B, and F–H), the *kinA*^E178K,E186K,K735R^-GFP mutant almost phenocopied the *kinA*^(1–894)^-GFP mutant: It formed a colony similar to that of the *kinA*^(1–894)^-GFP mutant (Fig. 3, A and B), and it also exhibited a strong hyphal-tip KinA-GFP accumulation (Fig. 3, F and G) and an abnormal early endosome accumulation (Fig. 3, F and H). As kinesin-1s are normally autoinhibited and their strong accumulation at cell periphery near microtubule plus ends has been linked to a loss of autoinhibition in different cell types (Baron et al., 2022; Kelliher et al., 2018; Seiler et al., 2000; Twelvetrees et al., 2016; Weijman et al., 2022), our results suggest strongly that the defect in dynein-mediated early endosome transport is caused by a loss of kinesin-1 autoinhibition.

### Autoinhibition is not essential for kinesin-1 to transport its cargoes

**Figure 4.**
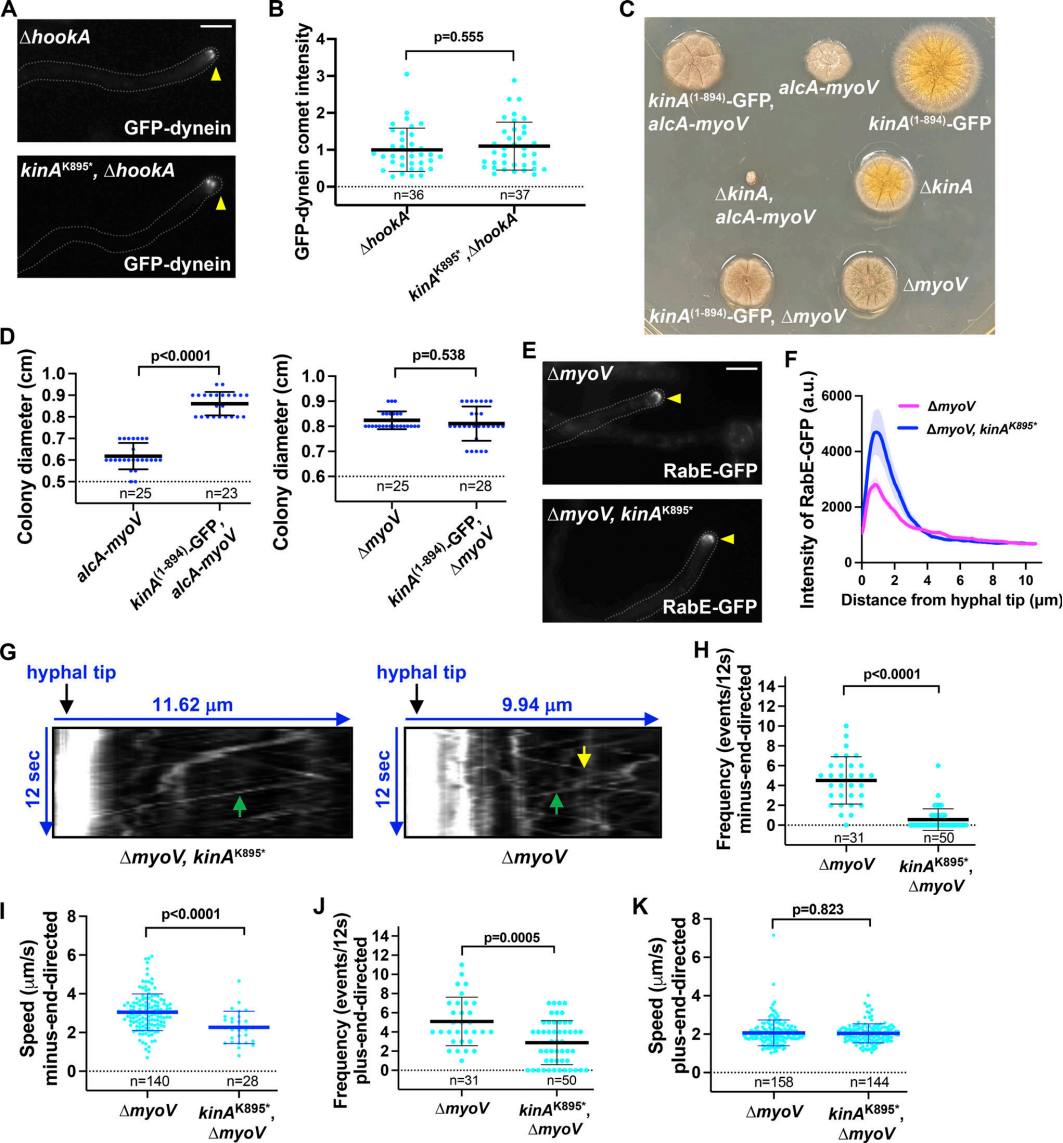
**Kinesin-1 autoinhibition is not critical for its own cargo-transport function. (A)** Microscopic images showing the hyphal-tip signals of GFP-dynein in the ∆*hookA* single and the *kinA*^K895^*, ∆*hookA* double mutant. Hyphal tip is indicated by a yellow arrowhead. Bar, 5 μm. **(B)** A quantitative analysis on GFP-dynein comet intensity in the ∆*hookA* single and the ∆*hookA*, *kinA*^K895^* double mutant. All values are relative to the average value for the ∆*hookA* single mutant, which is set as 1. Scatter plots with mean and SD values were generated by Prism 9. The Mann–Whitney test (unpaired, two tailed) was used for analyzing the two data sets without assuming normal distribution of the data. **(C)** Colony phenotypes of the various single and double myosin-V (*myoV*) and kinesin-1 (*kinA*) mutants. **(D)** Quantitative analyses of colony diameters of the *alcA-myoV* (*n* = 25) and the *kinA*^(1–894)^-GFP*, alcA-myoV* (*n* = 23) strains (unpaired, Mann–Whitney test) and of the ∆*myoV* (*n* = 25) and the *kinA*^(1–894)^-GFP, ∆*myoV* (*n* = 28) strains (unpaired, Mann–Whitney test). **(E)** Microscopic images showing the hyphal-tip signals of RabE-GFP in the ∆*myoV* single mutant and the ∆*myoV*, *kinA*^K895^* double mutant. Hyphal tip is indicated by a yellow arrowhead. Bar, 5 μm. **(F)** Line scans of RabE-GFP fluorescence intensity in the ∆*myoV* single mutant and the ∆*myoV*, *kinA*^K895^* double mutant (*n* = 20 for each strain). XY graphs with mean (solid lines) and SEM (shading) were generated by Prism 9. RabE-GFP intensity was significantly higher in the ∆*myoV*, *kinA*^K895^* double mutant than in the ∆*myoV* single mutant near the hyphal tip (between 0.65 and 1.43 μm from hyphal tip) (P < 0.0001, two-way ANOVA with Bonferroni’s multiple comparisons test). **(G)** Kymographs showing RabE-GFP signals (diagonal lines indicating movements of RabE-GFP-marked vesicles). Green arrows indicate plus-end-directed movements toward the hyphal tip, and a yellow arrow indicates minus-end-directed movement away from the hyphal tip. **(H)** A quantitative analysis on the frequency of minus-end-directed transport in the ∆*myoV* single mutant (*n* = 31 hyphal tips) and the ∆*myoV*, *kinA*^K895^* double mutant (*n* = 50 hyphal tips; unpaired, Mann–Whitney test, Prism 9). Scatter plots with mean and SD values were generated by Prism 9. **(I**) A quantitative analysis on the speed of minus-end-directed transport in the ∆*myoV* single mutant (*n* = 140 movements) and the ∆*myoV*, *kinA*^K895^* double mutant (*n* = 28 movements; unpaired, Mann–Whitney test, Prism 9). Scatter plots with mean and SD values were generated by Prism 9. **(J)** A quantitative analysis on the frequency of plus-end-directed transport in the ∆*myoV* single mutant (*n* = 31 hyphal tips) and the ∆*myoV*, *kinA*^K895^* double mutant (*n* = 50 hyphal tips; unpaired, Mann–Whitney test, Prism 9). Scatter plots with mean and SD values were generated by Prism 9. **(K)** A quantitative analysis on the speed of plus-end-directed transport in the ∆*myoV* single mutant (*n* = 158) and the ∆*myoV*, *kinA*^K895^* double mutant (*n* = 144) (unpaired, Mann–Whitney test, Prism 9). Scatter plots with mean and SD values were generated by Prism 9.

In the *kinA*^K895^* mutant, the overall intensity of GFP-dynein is higher than normal near the hyphal tip (Fig. 1 B), suggesting that dynein can be transported there but cannot leave. In the ∆*hookA* mutant lacking the early endosomal dynein adapter, dynein does not leave the plus end with its cargo, and its hyphal-tip intensity is higher than in wild type regardless of whether the *kinA*^K895^* mutation is present (Fig. S2 E). In the *kinA*^K895^*, ∆*hookA* double mutant, the plus-end dynein comet intensity is similar to that in the ∆*hookA* single mutant (Fig. 4, A and B), suggesting that kinesin-1 autoinhibition is not essential for the plus-end dynein accumulation. This is consistent with a previous notion that the posterior localization of dynein during Drosophila oogenesis needs kinesin-1 but not its IAK region for autoinhibition (Williams et al., 2014).

In filamentous fungi, kinesin-1 and myosin-V are both able to transport secretory vesicles to support hyphal tip extension (Pantazopoulou et al., 2014; Peñalva et al., 2017; Pinar et al., 2022; Schuchardt et al., 2005; Schuster et al., 2012). In *A. nidulans*, the ∆*kinA*, ∆*myoV*, or *alcA*-*myoV* (myosin-V conditional-null) single mutant forms a colony smaller than a wild-type colony (Requena et al., 2001; Taheri-Talesh et al., 2012; Zhang et al., 2011a). However, the ∆*kinA*, *alcA*-myoV double mutant is nearly inviable when grown on glucose that represses *myoV* expression (Zhang et al., 2011a; Fig. 4 C), and the ∆*kinA*, ∆*myoV* double mutant is also nearly inviable (Peñalva et al., 2017). In contrast, colony of the *kinA*^(1–894)^-GFP, *alcA*-*myoV* double mutant is even bigger than that of the *alcA-myoV* single mutant, and colony of the *kinA*^(1–894)^-GFP, ∆*myoV* double mutant is similar to that of the ∆*myoV* single mutant (Fig. 4 D). Despite the subtle difference in the genetic interactions involving the null allele verses the conditional-null allele of myosin-V, our data strongly indicate that kinesin-1 autoinhibition is not essential for the transporting function of kinesin-1 in supporting hyphal growth. Consistent with this notion, RabE (Rab11)-marked secretory vesicles, which are cargoes of kinesin-1 and myosin-V (Pantazopoulou et al., 2014; Peñalva et al., 2017; Pinar et al., 2022), reach the hyphal tip in the *kinA*^K895^*, ∆*myoV* double mutant (Fig. 4, E and F), and the hyphal-tip intensity of RabE-GFP is even higher in the double mutant than that in the ∆*myoV* mutant (Fig. 4 F).

RabE-marked secretory vesicles are cargoes of kinesin-1 and dynein (Pantazopoulou et al., 2014; Peñalva et al., 2017), although the cargo adapter has yet to be identified. These vesicles are normally tethered at the hyphal tip by the actin cytoskeleton, but in the ∆*myoV* background or upon loss of the actin cytoskeleton, their bidirectional movements can be observed (Pantazopoulou et al., 2014; Peñalva et al., 2017). In the *kinA*^K895^*, ∆*myoV* double mutant, both the frequency and the speed of dynein-mediated movement are significantly reduced compared to the ∆*myoV* single mutant (Fig. 4, G, H, and I). This differs from the result on early endosomes because only the frequency but not the speed of dynein-mediated early endosome movement is reduced in the *kinA*^K895^* mutant (Fig. 1, H and I). Possibly, as kinesin-1 drives the plus-end-directed movement of the RabE vesicles, its constitutive activation directly competes against dynein-mediated movement of the same cargo, and similar phenomena might occur in other systems (Baron et al., 2022; Kelliher et al., 2018). The frequency but not the speed of plus-end-directed RabE-vesicle movement is also lower in the presence of *kinA*^K895^* (Fig. 4, J and K), just like in the case of early endosomes whose plus-end-directed transport is driven by kinesin-3 rather than kinesin-1 (Fig. 1, J and K; Egan et al., 2012; Lenz et al., 2006; Wedlich-Söldner et al., 2002; Zekert and Fischer, 2009), most likely due to the lack of cargoes as they are being abnormally held at the hyphal tip.

### Kinesin-1 autoinhibition facilitates cargo adaptor-mediated dynein activation

**Figure 5.**
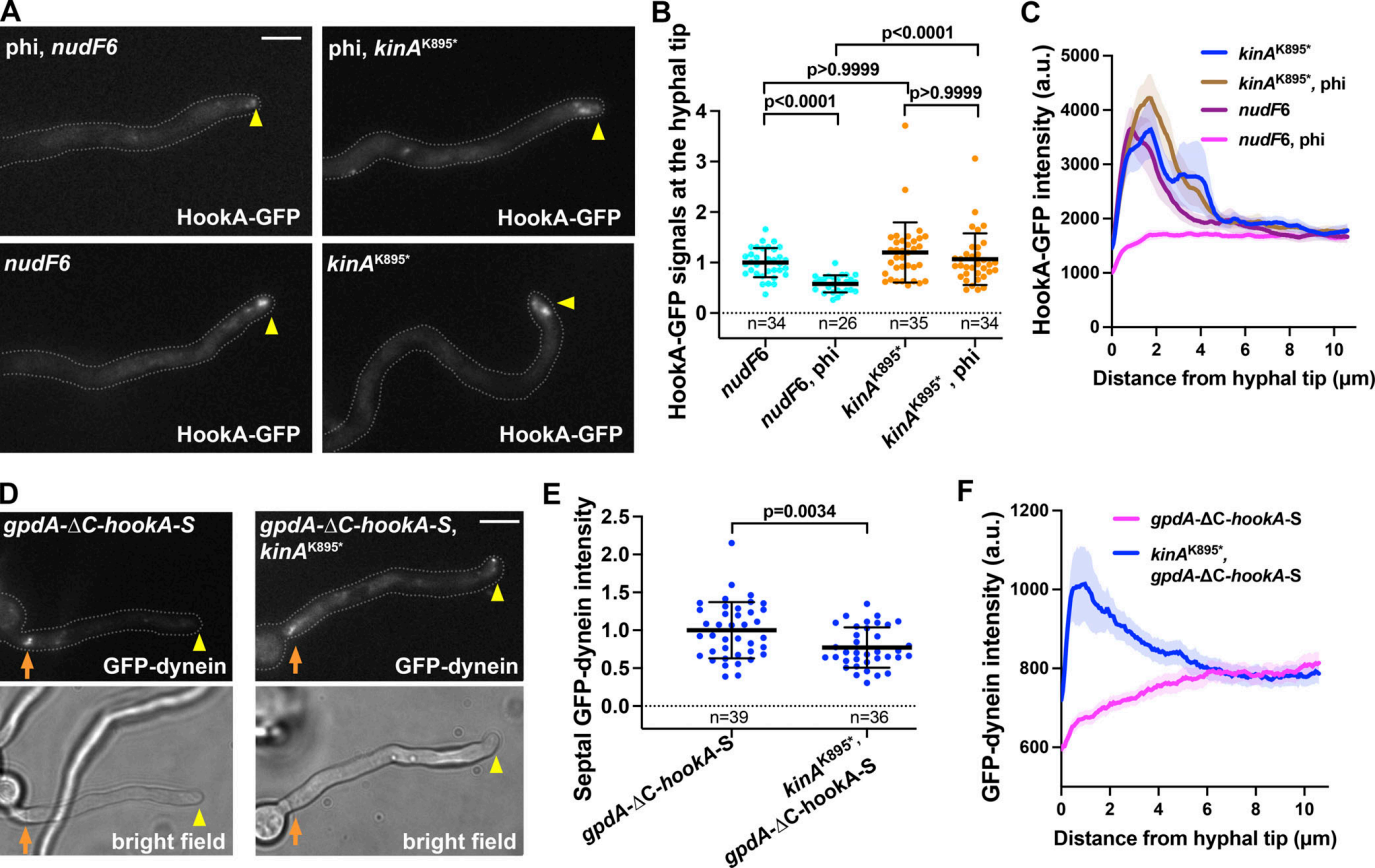
**The *kinA***^K895^* **mutation affects cargo-adapter-mediated dynein activation** in vivo**. (A)** Images of HookA-GFP in the *nudF*6 single mutant, the *nudF*6, phi double mutant, the *kinA*^k895^* single mutant and the *kinA*^k895^*, phi double mutant at 32°C (note that phi stands for *nudA*^R1602,K1645E^). Since we did the experiments at both 32°C (a semi-restrictive temperature for *nudF*6) and 37°C (a nearly restrictive temperature for *nudF*6), HookA-GFP was used as an early endosome marker since the HookA-GFP signals are clearer than those of mCherry-RabA at 37°C. Note that although the function of NudF/LIS1 is not completely lost at 32°C, the hyphal-tip accumulation of HookA-GFP signals is very obvious in the *nudF*6 single mutant. Hyphal tip is indicated by a yellow arrowhead. Bar, 5 μm. **(B)** A quantitative analysis on hyphal-tip accumulated HookA-GFP signals (Kruskal–Wallis with Dunn’s multiple comparisons test, unpaired). The average value for the *nudF*6 strain is set as 1. Scatter plots with mean and SD values were generated by Prism 9. **(C)** Line scans of HookA-GFP fluorescence intensity in the *nudF*6 single mutant, the *nudF*6, phi double mutant, the *kinA*^k895^* single mutant, and the *kinA*^k895^*, phi double mutant grown at 37°C. XY graphs with mean (solid lines) and SEM (shading) were generated by Prism 9. The intensity of HookA-GFP near the hyphal tip (between 0.39 and 1.82 μm from hyphal tip) was significantly different between the *nudF*6 single mutant and the *nudF*6, phi double mutant (P < 0.0001) but not significantly different between the *kinA*^k895^* single mutant and the *kinA*^k895^*, phi double mutant (*P* values are in between 0.192 and >0.999; two-way ANOVA with Tukey’s multiple comparisons test). **(D)** Dynein localization upon overexpression of the cargo adapter ∆C-HookA in a strain with wild-type *kinA* (*gpdA*-∆C-*hookA*-S) and in a strain with the *kinA*^K895^* mutation (*kinA*^K895^*, *gpdA*-∆C-*hookA*-S). Bright-field images are shown below to indicate hyphal shape and position of septum. Hyphal tip is indicated by a yellow arrowhead and septum by a brown arrow. Bar, 5 μm. **(E)** A quantitative analysis of dynein signals at septa (unpaired *t* test, two-tailed, Prism 9). All values are relative to the average value for the *gpdA*-∆C-*hookA*-S strain, which is set as 1. Scatter plots with mean and SD values were generated by Prism 9. **(F)** Line scans of GFP-dynein fluorescence intensity in the *gpdA*-∆C-*hookA*-S and the *kinA*^K895^*, *gpdA*-∆C-*hookA*-S strains. XY graphs with mean (solid lines) and SEM (shading) were generated by Prism 9. GFP-dynein intensity near the hyphal tip (between 0.26 and 1.69 μm from hyphal tip) was significantly higher in the *kinA*^K895^*, *gpdA*-∆C-*hookA*-S strain than in the *gpdA*-∆C-*hookA*-S strain (P < 0.0001, two-way ANOVA with Bonferroni’s multiple comparisons test, *n* = 30 hyphae for each strain).

In the *kinA*^K895^* mutant, we observed a decrease in the frequency but not the speed of dynein-mediated early endosome movement, which is conceptually similar to the defect observed in a mutant lacking NudF/LIS1 (although the defect in the LIS1 mutant is much more severe; Egan et al., 2012). LIS1 is a conserved dynein regulator that binds to the dynein motor domain and plays a specific role in dynein activation (Gillies et al., 2022; Markus et al., 2020; Reimer et al., 2022 *Preprint*). Dynein activation involves conformational changes of the dynein heavy chain dimer from an auto-inhibited “phi” conformation to the open conformation (Torisawa et al., 2014; Zhang et al., 2017a), and binding of dynactin and cargo adapter to the dynein tails turns dynein to a parallel conformation needed for processive motility (Zhang et al., 2017a). The phi mutation disrupts the phi structure and keeps dynein open, thereby facilitating dynein activation (Zhang et al., 2017a). In *A. nidulans*, the phi mutation (*nudA*^R1602,K1645E^) significantly reduces the abnormal hyphal-tip accumulation of early endosomes in the temperature-sensitive *nudF*6 (lis1) mutant (Fig. 5, A–C; Qiu et al., 2019), suggesting a role of LIS1 in overcoming the autoinhibited conformation (Qiu et al., 2019), similarly observed in other systems especially budding yeast (Elshenawy et al., 2020; Gillies et al., 2022; Htet et al., 2020; Marzo et al., 2020; Karasmanis et al., 2022 *Preprint*). However, the phi mutation does not significantly affect the hyphal-tip accumulation of early endosomes in the *kinA*^K895^* mutant (Fig. 5, A–C). Thus, kinesin-1 autoinhibition must be involved in a step of dynein-mediated transport beyond switching dynein to the open state.

Cargo adapters activate dynein to leave the microtubule plus ends both in vitro and in cultured cells (Baumbach et al., 2017; Jha et al., 2017; Lammers and Markus, 2015; McKenney et al., 2014; Qiu et al., 2019; Schlager et al., 2014; Splinter et al., 2012). In *A. nidulans*, overexpression of the cytosolic ∆C-HookA causes dynein to undergo a LIS1-dependent relocation from the microtubule plus ends to the minus ends on septa or spindle-pole bodies (Qiu et al., 2019). Since the spindle-pole body accumulation of activated dynein is cell-cycle dependent (Bieger et al., 2021), here we only focused on the septal minus ends (Zhang et al., 2017b). In the *kinA*^K895^* mutant, overexpression of ∆C-HookA causes the septal accumulation of dynein, but the plus-end-to-minus-end relocation is defective as evidenced by the more easily observable plus-end dynein comets and the reduced septal signals (Fig. 5, D–F). Thus, although kinesin-1 autoinhibition is not essential for cargo-adapter-mediated dynein activation, it facilitates this process.

Kinesin-mediated transport of dynein to the microtubule plus ends has been reported from fungi to mammalian neurons (although kinesin-7 instead of kinesin-1 is used in budding yeast; Carvalho et al., 2004; Roberts et al., 2014; Twelvetrees et al., 2016; Yamada et al., 2010; Zhang et al., 2003). In several systems, the plus-end accumulation of dynein and its regulator dynactin plays an important role in cargo binding or the initiation of cargo transport (Lenz et al., 2006; Lloyd et al., 2012; Markus and Lee, 2011; Moughamian and Holzbaur, 2012; Splinter et al., 2012; Vaughan et al., 2002; Xiang and Qiu, 2020). We suggest that kinesin-1 autoinhibition facilitates dynein-mediated transport in these systems. The initiation of dynein-mediated transport needs LIS1 and its binding protein NudE, as well as the dynactin complex and cargo adapters (Egan et al., 2012; Garrott et al., 2022; Lenz et al., 2006; Markus et al., 2020; Olenick and Holzbaur, 2019; Qiu et al., 2019; Reck-Peterson et al., 2018). Kinesin-1 autoinhibition facilitates this process even after dynein opening, but how it affects dynein activation by dynactin and cargo adapters still remains a mystery. Recent studies suggest that some activated dynein complexes contain not only two dynein dimers (Grotjahn et al., 2018; Urnavicius et al., 2018) but also two cargo adapters (Chaaban and Carter, 2022). One possibility is that uninhibited kinesin-1s fail to dissociate from dynein-dynactin after delivering them to the microtubule plus ends, which could interfere with an optimal dynein-dynactin-cargo adapter interaction needed for the initiation of cargo transport. Alternatively, the uninhibited kinesin-1s may bring dynein back to the microtubule plus ends near the hyphal tip shortly after initiation of a retrograde transport event (Gicking et al., 2022). These ideas would need to be further tested.

Recent studies indicate that an ALS-causing mutation of KIF5A is a gain-of-function mutation disrupting autoinhibition (Baron et al., 2022; Nakano et al., 2022; Pant et al., 2022). In *A. nidulans*, *kinA*^K895^* or *kinA*^(1–894)^-GFP is also a gain-of-function mutation affecting dynein-mediated early endosome transport in the presence of wild-type *kinA* (Fig. S2, F and G). As dynein defects are linked to ALS (Chevalier-Larsen and Holzbaur, 2006; Liu and Henty-Ridilla, 2022), a defect in dynein-mediated transport could possibly contributes to the KIF5A-mutation-caused ALS. It is worthwhile to test this idea and other potential factors including aggregation of uninhibited kinesins or possible changes of microtubules (Baron et al., 2022; Budaitis et al., 2022; Chiba et al., 2022; Kawano et al., 2022; Nakano et al., 2022; Pant et al., 2022).

## Materials and methods

### *A. nidulans* strains, media, and identification of the *eedE*16 (*kinA*^K895^*) mutation

*A. nidulans* strains used in this study are listed in Table S1. UV mutagenesis on spores of *A. nidulans* strains was done as previously described (Willins et al., 1995; Xiang et al., 1999). Specifically, we collected some asexual spores from colony of the XX222 strain with a pipet tip containing ∼100 μl of sterilized distilled water and added the spores to 30 ml of sterilized distilled water contained in a 50-ml tube. We then poured about 25 ml of spore suspension into a Petri dish and placed the dish ∼6 in under a Spectronics UV Lamp (ENF-240C) to be treated with the short wave ultraviolet (254 nm) for 5–10 min. Different volumes (for example, 1, 10, 50 or 100 μl) of both the untreated spore suspension and the treated spore suspension were spread on plates with solid rich medium and incubated at 37°C for 2 d. A typical mutagenesis should kill about 95% of the spores, which can be determined by counting the number of colonies. From the plates with colonies from the mutagenized spores, we selected colonies (normally about 1% of the total colonies) that looked smaller and less bright in color, which is indicative of a decrease in asexual spore production. Spores from these mutant colonies were cultured for further microscopic examination on early-endosome distribution and dynein localization. The *kinA*^K895^* mutant was obtained after we examined more than one hundred mutant colonies.

Solid rich medium was made of either YAG (0.5% yeast extract and 2% glucose with 2% agar) or YAG+UU (YAG plus 0.12% uridine and 0.11% uracil). Genetic crosses and diploid construction were done by standard methods. Solid minimal medium containing 1% glucose was used for selecting progeny from a cross and for selecting diploids. For live-cell imaging, cells were cultured in liquid minimal medium containing 1% glycerol for overnight at 32°C. All the biochemical analyses (for confirming strain genotypes) and genomic DNA preparation were done using cells grown at 32°C for overnight in liquid YG rich medium (0.5% yeast extract and 2% glucose). For experiments using strains containing the *alcA-nudA*^HC^ allele*,* we harvested spores from the solid minimal medium containing 1% glycerol and cultured them for imaging analysis in liquid minimal medium containing 1% glucose, which is a repressive medium for the *alcA* promoter. To identify the *eedE*16 mutation, we used whole-genome sequencing and bioinformatic service of Genewiz (www.genewiz.com), and we also used the software Integrative Genomics Viewer (IGV 2.4.3) to visualize the genomic sequencing data (Qiu et al., 2020).

### Construction of a strain containing the *kinA*-GFP fusion gene at the *kinA* locus

Strains were constructed using standard procedures used in *A. nidulans* (Nayak et al., 2006; Szewczyk et al., 2006; Yang et al., 2004). For constructing the *kinA*-GFP fusion, we used the following six oligos to amplify genomic DNA from RQ177 (Qiu et al., 2018) that contains the GFP-*AfpyrG* fusion (Yang et al., 2004): K5U (5′-AGT​CTT​TCA​GAG​ACG​CAG​GG-3′), BW2 (5′-TCG​TCT​ATC​AAA​AAA​CCA​ACT​TGT​G-3′), KF3 (5′-CAC​AAG​TTG​GTT​TTT​TGA​TAG​ACG​AGG​AGC​TGG​TGC​AGG​CG-3′), BW4 (5′-CCA​TCT​AGA​TAT​CTG​CAG​GAA​GGG​GCT​GTC​TGA​GAG​GAG​GCA​CTG-3′), BW5 (5′-CCC​CTT​CCT​GCA​GAT​ATC​TAG​ATG​G-3′) and BW6 (5′-GCT​GAA​GTT​GGT​TGA​TTT​GCG​G-3′). Specifically, K5U and BW2 were used to amplify the ∼1-kb fragment in the coding region, and BW5 and BW6 were used to amplify the ∼1-kb fragment in the 3′ untranslated region, and KF3 and BW4 were used to amplify the ∼2.7-kb GFP-*AfpyrG* fragment using genomic DNA from the RQ177 strain (Qiu et al., 2018). We then used two oligos, K5U and BW6, for a fusion PCR of the three fragments to generate the ∼4.6-kb *kinA*-GFP-*AfpyrG* fragment that we used to transform into the XY42 strain (Qiu et al., 2018) containing ∆*nkuA* (Nayak et al., 2006) and mCherry-RabA (Abenza et al., 2009; Zhang et al., 2010).

For transformation, spores from XY42 were cultured in a flask containing 50 ml YG+UU liquid medium, which was shaken overnight at 80 rpm at room temperature and then at 180 rpm at 32°C for about 1.5 h. The medium was poured off, and hyphae were then treated with about 20 ml solution containing cell-wall-lysing enzymes. This solution contains 10 ml of solution 1 (52.8 g of ammonium sulfate and 9.6 g of citric acid in 500 ml water, pH adjusted to 6.0 with 5 M KOH), 10 ml of solution 2 (5 g of yeast extract and 10 g of sucrose in 500 ml water), 0.25 ml of 1 M MgSO_4_, 200 mg of fraction V bovine serum albumin, 60 mg of lysing Enzymes (L1412; Sigma-Aldrich), and 0.05 ml of β-glucuronidase (G8885; Sigma-Aldrich). This mixture was made and filter-sterilized within 1 h before being used to treat the hyphae. After about 3 h of treatment with this solution at 32°C with shaking at 180 rpm, protoplasts were generated. The protoplasts were collected by centrifugation at 1,700 rpm for 1 min using a swing-bucket rotor (Eppendorf S-4-72), washed with 15 ml of ice-cold solution 3 (26.4 g of ammonium sulfate, 5 g of sucrose and 4.8 g of citric acid in 500 ml water, pH adjusted to 6.0 with 5 M KOH), and finally suspended in 0.5 ml of ice-cold solution 5 (4.48 g of KCl, 0.75 g of CaCl_2_ and 0.195 g of MES in 100 ml water, pH adjusted to 6.0 with 5 M KOH). In a 15-ml tube, 100 μl protoplast was mixed with 20 μl DNA (1–2 μg total) and 50 μl ice-cold solution 4 (25 g of PEG 6000 or 8000 (P2139; Sigma-Aldrich), 1.47 g of CaCl_2_.2H_2_O, 4.48 g of KCl, and 1.0 ml of 1 M Tris-HCl pH 7.5 in 100 ml water). This mixture was kept on ice for 20 min, followed by addition of 1 ml solution 4 with gentle mixing. The tube was kept at room temperature for 20 min. 10 ml of 50°C pre-melted solid medium (YAG + 0.6 M KCl) was added into the tube and the mixture was poured into a petri dish with a thin layer of the same solid medium (YAG + 0.6 M KCl). After the plates were incubated at 37°C for 2–3 d, colonies of transformants appeared. Autoclaved toothpicks were used to touch the top of the individual colony and transfer the asexual spores onto a YAG plate, which was incubated at 37°C for 2 d. The transformants were then screened by microscopically observing the GFP signals, and the presence of the *kinA*-GFP fusion was confirmed by western blotting analysis with a polyclonal anti-GFP antibody from Clontech. In addition, we also performed a diagnostic PCR to verify the correct integration using oligos K5UTR (5′-GAA​CGA​CCT​CAC​AGA​CTC​A-3′) and AFpyrG3 (5′-GTT​GCC​AGG​TGA​GGG​TAT​TT-3′).

### Construction of a strain containing the *kinA*^(1–894)^-GFP allele at the *kinA* locus

For constructing the *kinA*^(1–894)^-GFP strain, we made the *kinA*^(1–894)^-GFP-*AfpyrG* fragment by inserting the GFP-*AfpyrG* fragment into the *kinA*^K895^* mutation site right before the stop codon. The following four oligos were used to make the *kinA*^(1–894)^-GFP-*AfpyrG* construct: K5U, K895.R (5′-GCA​CCA​GCT​CCA​GCG​ATT​CGG​GAG​CCA​G-3′), K895.F (5′-AAT​CGC​TGG​AGC​TGG​TGC​AGG​CG-3′), and BW6. Specifically, K5U and K895.R were used to amplify a ∼0.9-kb fragment of the 5′ coding region, and K895.F and BW6 were used to amplify a ∼3.6-kb fragment using genomic DNA from the RQ197 strain (containing *kinA*-GFP) as template. We then used two oligos, K5U and BW6, for a fusion PCR to fuse the two fragments to generate the ∼4.5-kb *kinA*^(1–894)^-GFP-*AfpyrG* fragment that we used to transform the XY42 strain and the RQ54 strain. The transformants were screened by microscopically observing the GFP signals and further confirmed by a Western blotting analysis with a polyclonal anti-GFP antibody from Clontech. In addition, we also performed a diagnostic PCR to verify the correct integration using oligos K5UTR (5′-GAA​CGA​CCT​CAC​AGA​CTC​A-3′) and GFP-5R (5′-CAG​TGA​AAA​GTT​CTT​CTC​CTT​TAC​T-3′).

### Construction of a strain containing the *kinA*^∆IAK^-GFP allele at the *kinA* locus

We made the *kinA*^∆IAK^-GFP-*AfpyrG* fragment using the following four oligos: K5U2 (5′-GCC​AGT​CTT​TCA​GAG​ACG​CAG​G-3′), IAK-R (5′-ACG​GAG​AGG​TCG​GGA​GCC​AGC​GAA​GCT-3′), IAK-F (5′-GGC​TCC​CGA​CCT​CTC​CGT​GGC​GGC​G-3′), and Kin3R (5′-GCT​GAA​GTT​GGT​TGA​TTT​GCG​GAC-3′). Specifically, K5U2 and IAK-R were used to amplify a ∼0.9-kb fragment of the 5′ coding region, and IAK-F and Kin3R were used to amplify the ∼3.7-kb fragment using genomic DNA from the RQ197 strain (containing *kinA*-GFP) as template. We then used two oligos, K5U2 and Kin3R, for a fusion PCR to fuse the two fragments to generate the ∼4.6-kb *kinA*^∆IAK^-GFP-*AfpyrG* fragment that we used to transform the XY42 strain. The transformants were screened by microscopically observing the GFP signals. In addition, we also performed a diagnostic PCR to verify the correct integration using oligos K5U3 (5′-GTT​TTC​TGA​CAA​CGA​GCG​GAA​GC-3′) and GFP5R2 (5′-GCA​TCA​CCT​TCA​CCC​TCT​CCA​C-3′), which amplified a product of ∼1.3-kb. This 1.3-kb fragment was sequenced to verify the deletion of the IAK-coding sequence in the absence of other mutations. The sequence primers used were K5U3 and GFP5R2.

### Construction of a strain containing the *kinA*^K895E^-GFP allele at the *kinA* locus

We made the *kinA*^K895E^-GFP-*AfpyrG* fragment using the following four oligos: K5U, K895ER (5′-CCG​CCA​CGG​AGA​GGC​TCA​GCG​ATT​CGG​GAG​CCA​GC-3′), K895EF (5′-GAG​CCT​CTC​CGT​GGC​GG-3′), and BW6. Specifically, K5U and K895ER were used to amplify a ∼0.9-kb fragment of the 5′ coding region, and K895EF and BW6 were used to amplify the 3.7-kb fragment using genomic DNA from the RQ197 strain (containing *kinA*-GFP) as template. We then used two oligos, K5U and Kin3R, for a fusion PCR to fuse the two fragments to generate the ∼4.6-kb *kinA*^K895E^-GFP-*AfpyrG* fragment that we used to transform the XY42 strain. The transformants were screened by microscopically observing the GFP signals. In addition, we also performed a diagnostic PCR to verify the correct integration using oligos K5U3 and GFP5R2, which amplified a product of ∼1.3-kb. This 1.3-kb fragment was sequenced to verify the K895E mutation in the absence of other mutations. The sequence primers used were K5U3 and GFP5R2.

### Construction of a strain containing the *kinA*^E186K^-GFP allele at the *kinA* locus

We made the *kinA*^E186K^-GFP-*AfpyrG* fragment using the following four oligos: K5UTR2 (5′-TGA​ACG​ACC​TCA​CAG​ACT​CAC​TCC-3′), E186K-R (5′-TGA​CTT​CGT​AAA​CCT​TTT​GTA​CGC​TAG​AGA​C-3′), E186K-F (5′-GTC​TCT​AGC​GTA​CAA​AAG​GTT​TAC​GAA​GTC​A-3′), and KinR1 (5′-GCT​TCC​GCT​CGT​TGT​CAG​AAA​AC-3′). Specifically, K5UTR2 and E186K-R were used to amplify a ∼0.9-kb fragment, and E186K-F and KinR1 were used to amplify a ∼1.2-kb fragment using genomic DNA from the RQ197 strain (containing *kinA*-GFP) as template. We then used two oligos, K5UTR2 and KinR1, for a fusion PCR to fuse the two fragments to generate a ∼2.1-kb fragment. We co-transformed the ∼2.1-kb fragment with the ∼4.6-kb *kinA*-GFP-*AfpyrG*-containing fragment (amplified using K5U2 and Kin3R as primers) into the XY42 strain. The transformants were screened by microscopically observing the GFP signals. In addition, we also performed a PCR using K5UTR3 (5′-CAG​CTC​CGT​ATC​TCT​TGT​CGG​TCT-3′) and GFP5R2 as primers, and sequenced the PCR product with primers K5UTR2, KinR1, K5U3, and GFP5R2 to verify the E186K mutation in the absence of other mutations.

### Construction of a strain containing the *kinA*^E178K,P426L^-GFP allele at the *kinA* locus

We made the *kinA*^E178K^-GFP-*AfpyrG* fragment using the following four oligos: K5UTR2, E178K-R (5′-CTA​GAG​ACG​TAA​ACC​TTA​AGC​AGA​CCT​TTG​A-3′), E178K-F (5′-TCA​AAG​GTC​TGC​TTA​AGG​TTT​ACG​TCT​CTA​G-3′), and KinR1. Specifically, K5UTR2 and E178K-R were used to amplify a ∼0.9-kb fragment, and E178K-F and KinR1 were used to amplify a ∼1.2-kb fragment using genomic DNA from the RQ197 strain (containing *kinA*-GFP) as template. We then used two oligos, K5UTR2 and KinR1, for a fusion PCR to fuse the two fragments to generate a ∼2.1-kb fragment. We co-transformed the ∼2.1-kb fragment with the ∼4.6-kb *kinA*-GFP-*AfpyrG*-containing fragment (amplified using K5U2 and Kin3R as primers) into the XY42 strain. The transformants were screened by microscopically observing the GFP signals. In addition, we also performed a PCR using K5UTR3 and GFP5R2 as primers, and sequenced the PCR product with primers K5UTR2, KinR1, K5U3, and GFP5R2 to verify the E178K mutation in the absence of other mutations. However, the sequencing analysis detected the unintended P426L mutation.

### Construction of a strain containing the *kinA*^E178K,E186K,K735R^-GFP allele at the *kinA* locus

We made the *kinA*^E178K,E186K^-GFP-*AfpyrG* fragment using the following four oligos: K5UTR2, E178K-R, E178K-F, and KinR1. Specifically, K5UTR2 and E178K-R were used to amplify a ∼0.9-kb fragment using genomic DNA from the RQ400 strain (containing *kinA*^E178K, P426L^-GFP) as template, and E178K-F and KinR1 were used to amplify a ∼1.2-kb fragment using genomic DNA from the RQ395 strain (containing *kinA*^E186K^-GFP) as template. We then used two oligos, K5UTR2 and KinR1, for a fusion PCR to fuse the two fragments to generate a ∼2.1-kb fragment. We co-transformed the ∼2.1-kb fragment with the ∼4.6-kb *kinA*-GFP-*AfpyrG*-containing fragment (amplified using K5U2 and Kin3R as primers) into the XY42 strain. The transformants were screened by microscopically observing the GFP signals. In addition, we also performed a PCR using K5UTR3 and GFP5R2 as primers, and sequenced the PCR product with primers K5UTR2, KinR1, K5U3, and GFP5R2 to verify the E178K and E186K mutations to verify the E178K and E186K mutations in the absence of other mutations. However, the sequencing analysis detected the unintended K735R mutation.

### Live-cell imaging and analyses

Images were captured using an Olympus IX73 inverted fluorescence microscope linked to a PCO/Cooke Corporation Sensicam QE cooled CCD camera and controlled by the IPlab software. An UPlanSApo 100× objective lens (oil) with a 1.40 numerical aperture (NA) was used. A filter wheel system with GFP/mCherry-ET Sputtered series with high transmission (Biovision Technologies) was used. Images used for line scans and those for Fig. 2 E, Fig. 4, G–K, and Fig. S2 A were generated using a Nikon Ti2-E inverted microscope with Ti2-LAPP motorized Total Internal Reflection Fluorescence (TIRF) module and a Chromatic Aberration Free Infinity (CFI) apochromat TIRF 100 x 1.49 NA objective lens (oil). The microscope was controlled by NIS-Elements software using 488 and 561 nm lines of LUN-F laser engine and ORCA-Fusion BT cameras (Hamamatsu). For all images, cells were grown in the LabTek Chambered #1.0 borosilicate coverglass system (Nalge Nunc International). Images were taken at room temperature immediately after the cells were taken out of the incubators. Cells were cultured overnight in minimal medium with 1% glycerol and supplements at 32°C. The IPLab software (for the Olympus IX73 microscope) or the NIS-Elements software (for the Nikon Ti2-E microscope) was used for image acquisition and analysis. Image labeling was done using Microsoft PowerPoint and/or Adobe Photoshop. For quantitation of dynein comet signal intensity, a region of interest (ROI) was selected, and the IPLab program was used to measure the total signal intensity (Sum) within the ROI. The ROI box was then dragged to a nearby area outside of the cell to take the background value, which was then subtracted from the intensity value. For quantitation of HookA-GFP-marked early endosome accumulation at the hyphal tip, the total hyphal-tip signals (Sum) were measured, and background subtracted the same way. For quantitation of septal dynein signals, a region of interest (ROI) was selected and the IPLab program was used to measure the maximal signal intensity (Max) within the ROI. The ROI box was then dragged to a nearby area outside of the cell to take the background value, which was then subtracted from the intensity value. Hyphae were chosen randomly as long as the hyphal tip segment looked focused. For measuring the signal intensity of a microtubule plus-end comet formed by GFP-dynein, only those comets close to the hyphal tip were measured. For measuring GFP-dynein signal intensity at septa, usually only the septum most proximal to the hyphal tip was measured. For line scans, we draw a line starting from the hyphal tip in the middle of the hypha and get the average intensity value for the width of 2 μm, which is normally the hyphal width.

### Statistical analysis

All statistical analyses were done using GraphPad Prism 9 for macOS (version 9.4.1, 2022). The D’Agostino & Pearson normality test was performed on all data sets (except for the percentage data presented in Fig. 3, E and H and Fig. S2 G where n is 3). For data sets that passed the normality test, *t* test (unpaired, two tailed) was used for analyzing two data sets and ordinary one-way ANOVA (unpaired) for multiple data sets. For data sets that did not pass the normality test, non-parametric tests were used without assuming Gaussian distribution. Specifically, Mann–Whitney test (unpaired, two tailed) was used for analyzing two data sets and the Kruskal–Wallis ANOVA test (unpaired) with Dunn’s multiple comparisons test was used for analyzing multiple data sets. Note that adjusted P values were generated from the Kruskal–Wallis ANOVA test with Dunn’s multiple comparisons test. For the percentage data presented in Fig. 3, E and H; and Fig. S2 G where *n* is 3, which is too small for the normality test, we used ordinary one-way ANOVA (unpaired) for statistical analysis. For analyzing the line scans of two groups, we used two-way ANOVA with Bonferroni’s multiple comparisons test to compare each cell mean with other cell mean in that row (Note that each row represents a position with a particular distance from the hyphal tip). For analyzing the line scans of multiple groups, we used two-way ANOVA with Tukey’s multiple comparisons test to compare each cell mean with other cell mean in that row.

### Online supplemental material

Fig. S1 presents a protein sequence alignment of three different kinesin-1s. Fig. S2 shows phenotypes of the *kinA*^K895^* mutant and various diploids. Video 1 shows microtubules labeled by GFP-TubA in a wild-type hypha. Video 2 shows microtubules labeled by GFP-TubA in a *kinA*^K895^* mutant hypha. Table S1 lists *A. nidulans* strains used in this study.

## Supplementary Material

Table S1 shows *A. nidulans* strains used in this study.
