# Supplementary material for: Kinesin-1 autoinhibition facilitates the initiation of dynein cargo transport
Source: J Cell Biol. 2022 Dec 16;222(3):e202205136. doi: 10.1083/jcb.202205136 (PMC9802684; doi:10.1083/jcb.202205136)
Supplement: Table S1 — shows A. nidulans strains used in this study. [file JCB_202205136_TableS1.docx]

**Supplemental Table 1. *Aspergillus nidulans* strains used in this study**

| **Strain** | **Genotype** | **Source** |
| --- | --- | --- |
| *alcA-myoV* | *alcA*-GFP-*myoV*-*pyr4*; *pyrG*89; *pyroA*4; *wA*3 | (Zhang et al., 2011a) |
| JZ504 | GFP-*nudA*^HC^; *∆hookA-AfpyrG; argB*2*::[argB*-alcAp*::mCherry*-RabA]*; *pyroA*4 | (Qiu et al., 2021) |
| LO1830 | *∆myoV*::*AfpyrG;* *pyrG*89; *pyroA*4; *riboB*2; ∆*nkuA::argB*  *(*Note: *myoV* was called *myoE* in the original publication*)* | (Taheri-Talesh et al., 2012) |
| RQ2 | GFP-*nudA*^HC^*; argB*2*::[argB*-alcAp*::mCherry*-RabA];* ∆*nkuA::argB; pyrG*89; *pyroA*4; *yA*2 | (Qiu et al., 2013) |
| RQ54 | *argB*2*::[argB*-alcAp*::mCherry*-RabA];* ∆*nkuA::argB; pyrG*89; *pyroA*4; *wA*2 | (Qiu et al., 2013) |
| RQ102 | GFP-*nudA*^HC^; *∆hookA-AfpyrG; argB*2*::[argB*-alcAp*::mCherry*-RabA]*; *pantoB100*; *yA2* | (Qiu et al., 2021) |
| RQ177 | p25^K4A/K7G/E9A/Y10G/E12A (N-AG)^-GFP-*AfpyrG ;* *argB*2*::[argB*-alcAp*::mCherry*-RabA]*; ∆*nkuA::argB; pyrG*89*; pantoB100*; *yA2* | (Qiu et al., 2018) |
| RQ287 | GFP-*nudA*^HC^; *gpdA*-∆C-*hookA*-S-*AfpyrG; yA*2 | (Qiu et al., 2019) |
| XX213 | ∆*kinA*-*pyr*4; GFP-*nudA*^HC^*; argB*2*::[argB*-alcAp*::mCherry*-RabA]* | (Zhang et al., 2010) |
| XX222 | GFP-*nudA*^HC^*; argB*2*::[argB*-alcAp*::mCherry*-RabA]*; *yA2*; *pantoB*100 | (Zhang et al., 2010) |
| XY42 | *argB*2*::[argB*-alcAp*::mCherry*-RabA]*; *∆nkuA::argB*; *pyrG*89; *yA2*; *pantoB*100 | (Qiu et al., 2018) |
| Mu16 or *eedE*16 | *kinA*^K895^*; GFP-*nudA*^HC^*; argB*2*::[argB*-alcAp*::mCherry*-RabA]*; *yA2*; *pantoB*100 | This work |
| RQ197 | *kinA*-GFP-*AfpyrG*; *argB*2*::[argB*-alcAp*::mCherry*-RabA]*; *∆nkuA::argB*; *pyrG*89; *yA2*; *pantoB*100 | This work |
| RQ358 | *kinA*^(1-894)^-GFP-*AfpyrG*; *argB*2*::[argB*-alcAp*::mCherry*-RabA]*; *∆nkuA::argB*; *pyrG*89; *yA2*; *pantoB*100 | This work |
| RQ361 | *kinA*^(1-894)^-GFP-*AfpyrG*; *alcA-nudA*^HC^*-pyr4*; *argB*2*::[argB*-alcAp*::mCherry*-RabA]*; possibly *∆nkuA::argB*; possibly *pyrG*89 | This work |
| RQ391 | *kinA*^∆IAK^-GFP-*AfpyrG*; *argB*2*::[argB*-alcAp*::mCherry*-RabA]*; *∆nkuA::argB*; *pyrG*89; *yA2*; *pantoB*100 | This work |
| RQ392 | *kinA*^K895E^-GFP-*AfpyrG*; *argB*2*::[argB*-alcAp*::mCherry*-RabA]*; *∆nkuA::argB*; *pyrG*89; *yA2*; *pantoB*100 | This work |
| RQ395 | *kinA*^E186K^-GFP-*AfpyrG*; *argB*2*::[argB*-alcAp*::mCherry*-RabA]*; *∆nkuA::argB*; *pyrG*89; *yA2*; *pantoB*100 | This work |
| RQ398 | *kinA*^E178K,E186K,K735R^-GFP-*AfpyrG*; *argB*2*::[argB*-alcAp*::mCherry*-RabA]*; *∆nkuA::argB*; *pyrG*89; *yA2*; *pantoB*100 | This work |
| RQ400 | *kinA*^E178K,P426L^-GFP-*AfpyrG*; *argB*2*::[argB*-alcAp*::mCherry*-RabA]*; *∆nkuA::argB*; *pyrG*89; *yA2*; *pantoB*100 | This work |
| XX367 | *hhoA*(histone H1)-GFP-*AfriboB*; *argB*2*::[argB*-alcAp*::mCherry*-RabA]* | This work |
| XX679 | *kinA*^K895^*; GFP-*nudA*^HC^; *gpdA*-∆C-*hookA*-S-*AfpyrG* | This work |
| XX711 | *kinA*(^1-894)^-GFP-*AfpyrG*; *alcA*-GFP-*myoV*-*pyr4*; *pyroA*4; *wA*2; possibly *∆nkuA::argB*; possibly *pyrG*89 | This work |
| XX713 | *alcA*-GFP-*myoV*-*pyr4*; *pyroA*4; *wA*2; possibly *∆nkuA::argB*; possibly *pyrG*89 | This work |
| XX716 | GFP-*nudA*^HC^*; ∆hookA-AfpyrG; argB*2*::[argB*-alcAp*::mCherry*-RabA]*; w*A2* | This work |
| XX718 | *kinA*^K895^*; GFP-*nudA*^HC^*; ∆hookA-AfpyrG; argB*2*::[argB*-alcAp*::mCherry*-RabA]*; w*A2* | This work |
| XX738 | *kinA*^K895^*; *argB*2*::[argB*-alcAp*::mCherry*-RabA]*; *pyrG*89; *wA2;* possibly ∆*nkuA::argB* | This work |
| XX752 | *kinA*^(1-894)^-GFP-*AfpyrG*; *∆myoV*::*AfpyrG;* *pyrG*89; ∆*nkuA::argB; yA2* | This work |
| XX782 | *∆myoV*::*AfpyrG;* *argB*2*::[argB*-alcAp*::mCherry*-RabA];* possibly *pyrG*89; possibly ∆*nkuA::argB* | This work |
| XX810 | diploid XX738/RQ197 | This work |
| XX811 | diploid RQ54/RQ197 | This work |
| XX814 | diploid RQ54/RQ358 | This work |
| XX824 | *∆myoV*::*AfpyrG;* *rabE*p::GFP-RabE::*AfpyrG*::*rabE*; *argB*2*::[argB*-alcAp*::mCherry*-RabA]; pyrG*89; possibly ∆*nkuA::argB* | This work |
| XX825 | *kinA*^K895^*; *∆myoV*::*AfpyrG;* *rabE*p::GFP-RabE::*AfpyrG*::*rabE*; *argB*2*::[argB*-alcAp*::mCherry*-RabA]; pyrG*89; possibly ∆*nkuA::argB* | This work |
| XX828 | diploid XX738/RQ358 | This work |
| XX834 | *hookA*-GFP-*afpyrG*; *kinA*^K895^*; *argB*2*::[argB*-alcAp*::mCherry*-RabA];* possibly *pyrG*89; possibly ∆*nkuA::argB* | This work |
| XX835 | *hookA*-GFP-*afpyrG*; *kinA*^K895^*; possibly *pyrG*89; possibly ∆*nkuA::argB* | This work |
| XX836 | *nudA*^R1602,K1645E^; *hookA*-GFP-*afpyrG*; *nudF*6; possibly *pyrG*89; possibly ∆*nkuA::argB* | This work |
| XX838 | *hookA*-GFP-*afpyrG*; *nudF*6; possibly *pyrG*89; possibly ∆*nkuA::argB* | This work |
| XX840 | *nudA*^R1602,K1645E^; *hookA*-GFP-*afpyrG*; *kinA*^K895^*; possibly *pyrG*89; possibly ∆*nkuA::argB* | This work |
| XX842 | *nudA*^R1602,K1645E^; *hookA*-GFP-*afpyrG*; *kinA*^K895^*; *argB*2*::[argB*-alcAp*::mCherry*-RabA];* possibly *pyrG*89; possibly ∆*nkuA::argB* | This work |
| XX847 | *kinA*^K895^*; *hhoA*(histone H1)-GFP-*AfriboB*; *argB*2*::[argB*-alcAp*::mCherry*-RabA]* | This work |
| XX868 | *kinA*^K895^*; GFP-*tubA*; Δ*yA*::NLS-DsRed; *pyrG*89 | This work |
| XX873 | GFP-*tubA*; Δ*yA*::NLS-DsRed; *pyrG*89; *pantoB*100 | This work |
| XX898 | *kinA*^K895^*; *alcA*-GFP-*tubA*-*pyr*4; *ClipA*-mCherry::*AfpyroA; wA*2 | This work |
| XX899 | *alcA*-GFP-*tubA*-*pyr*4; *ClipA*-mCherry::*AfpyroA; wA*2 | This work |
